# Hepatic and intestinal microcirculation and pulmonary inflammation in a model of veno-venous extracorporeal membrane oxygenation in the rat

**DOI:** 10.1186/s40635-025-00824-x

**Published:** 2025-11-19

**Authors:** Fabian Edinger, Thomas Zajonz, Nico Mayer, Goetz Schmidt, Emmanuel Schneck, Michael Sander, Christian Koch

**Affiliations:** Department of Anaesthesiology, Critical Care Medicine and Pain Therapy, University Hospital of Giessen, Justus-Liebig-University, Rudolf-Buchheim-Str.7, 35392 Giessen, Germany

**Keywords:** V–V ECMO, Inflammation, Intestinal microcirculation, Hepatic microcirculation

## Abstract

**Background:**

Veno-venous (V–V) extracorporeal membrane oxygenation (ECMO) is widely used in critical care but remains associated with high mortality rates (22–68%). In septic shock, increased pulmonary inflammation and impaired intestinal and hepatic microcirculation have been observed during ECMO therapy. To explore the impact of ECMO-induced inflammation, this study used a rat model with varying ECMO blood flows to assess intestinal and hepatic microcirculation and lung inflammation.

**Methods:**

Thirty male Lewis rats were randomised into three groups: sham, low-flow ECMO (60 mL/kg/min), and high-flow ECMO (90 mL/kg/min). V–V ECMO was established via femoral drainage and jugular return. Microcirculation in the intestine and liver was measured using micro-light guide spectrophotometry after laparotomy. Systemic and pulmonary inflammation were evaluated through cytokine levels in plasma and bronchoalveolar lavage (BAL), focusing on tumour necrosis factor-alpha (TNF-α), interleukins 6 (IL6) and 10 (IL10), and C–X–C motif chemokine ligands 2 (CXCL2) and 5 (CXCL5). Hemodynamic data were obtained using a left ventricular pressure–volume catheter.

**Results:**

Intestinal oxygenation was significantly impaired only during low-flow ECMO therapy (65% [62–70%]) compared to sham therapy (76% [72–79%], *p* = 0.003), while hepatic microcirculation was reduced during both low-flow (21% [14–26%]) and high-flow (19% [16–21%]) ECMO therapy compared to sham therapy (43% [38–48%], all *p* < 0.001). Serum TNF-α levels were only significantly elevated during high-flow ECMO therapy (1 h: 14 [12–22] pg/mL; 2 h: 18 [15–38] pg/mL) compared to the sham procedure (1 h: 10 [9–11] pg/mL; 2 h: 10 [9–11] pg/mL; *p* = 0.033). In contrast, BAL IL6 levels were significantly lower during both high- and low-flow ECMO therapy (32 pg/mL) than sham therapy (81 pg/mL, *p* ≤ 0.001). IL10, CXCL2, and CXCL5 levels did not differ significantly between the low- and high-flow ECMO and sham therapies.

**Conclusions:**

ECMO-induced inflammation is blood flow dependent. In healthy rats, high-flow ECMO did not impair intestinal microcirculation and was associated with reduced pulmonary inflammation, likely due to lung-protective ventilation.

## Background

While the first prolonged therapy of a patient with acute respiratory distress syndrome using veno-venous (V–V) extracorporeal membrane oxygenation (ECMO) was published in 1972, V–V ECMO therapy was performed only in “well-selected patients” for a long time [1]. During the last two decades, the application of V–V ECMO has increased continuously due to supporting evidence from the CESAR and EOLIA trials [2, 3]. The two large respiratory virus pandemics (influenza in 2009 and severe acute respiratory syndrome coronavirus 2 in 2020) have also increased clinical experience with V–V ECMO [4, 5]. Nonetheless, mortality rates of 22–68% have been reported [6, 7]. A recent meta-analysis demonstrated that V–V ECMO therapy was associated with improved 60-day and 1-year mortality but increased intensive care unit mortality [8].

It has to be noted that ECMO therapy is associated with an ECMO-induced inflammation due to the large foreign surface of the membrane and the circuit and its interaction with the endothelia [9]. Since inflammatory reactions are difficult to compare in humans due to the heterogeneity among critically ill patients, models of V–V ECMO performed with inbred rat strains offer the opportunity to produce reproducible results. In addition to our results in rats treated with veno-arterial ECMO, our group has demonstrated ECMO-induced inflammation during V–V ECMO therapy [10, 11]. Notably, during V–V ECMO therapy, highly oxygenated blood is pumped through hypoxic lungs. It is well-understood that hyperoxia is associated with the production of reactive oxygen species, which are known to induce pulmonary cell death [12]. Hyperoxaemia is associated with increased mortality during severe infections [12]. In our previous studies, we showed increased pulmonary inflammation in rats with septic shock during V–V ECMO therapy. However, the effect of highly oxygenated blood on the lungs of rats without septic shock during V–V ECMO is unclear.

While no differences in plasma cytokine levels were observed, rats with septic shock presented with reduced intestinal and hepatic microcirculation during V–V ECMO therapy compared to rats with septic shock without V–V ECMO support [13]. However, the impact of ECMO-induced inflammation on the intestinal and hepatic microcirculation in the absence of septic shock during V–V ECMO with femoral drainage and jugular return is unknown.

Therefore, the primary aim of this study was to investigate the intestinal and hepatic microcirculation in a model of V–V ECMO in healthy rats.

The secondary aim was to evaluate the inflammatory response in the lungs during V–V ECMO therapy.

## Results

### Intestinal and hepatic microcirculation

**Fig. 1:**
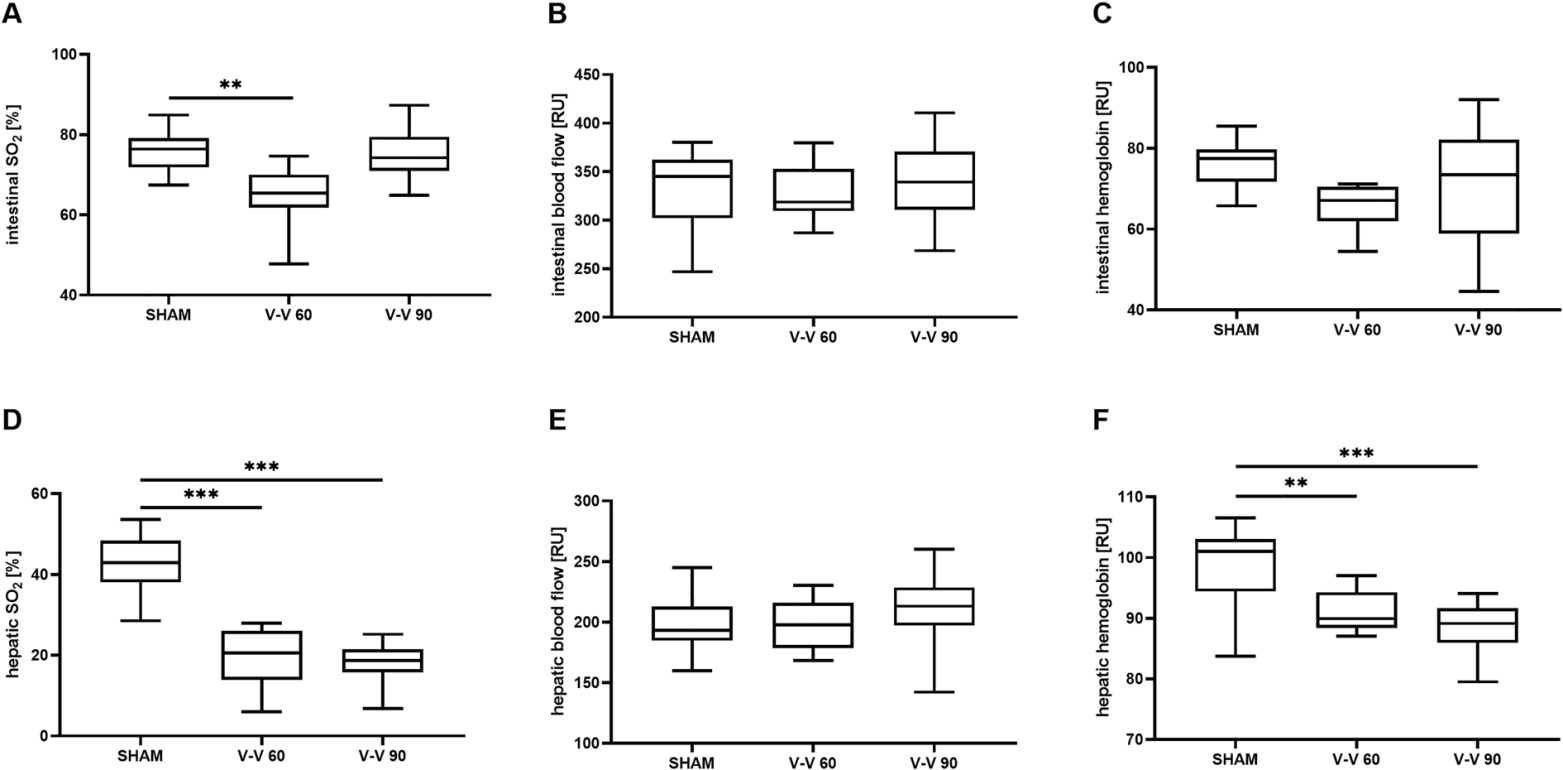
Means of the time-course of intestinal. **A** regional oxygen saturation, **B** relative blood flow, and **C** relative haemoglobin concentration, as well as **D** hepatic regional oxygen saturation, **E** relative blood flow, and **F** relative haemoglobin concentration. Intestinal regional oxygen saturation was lower during V–V 60 therapy than sham therapy. Hepatic regional oxygen saturation and relative haemoglobin concentration were impaired in all animals during ECMO therapy compared to sham therapy (*n* = 10/group). The asterisks denote the degree of statistical significance: **, *p* < 0.01; ***, *p* < 0.001. Box and whisker plots show the median, interquartile range (box), and minimum and maximum (whiskers). *ECMO* extracorporeal membrane oxygenation, *RU* relative units, *SO*_*2*_ tissue oxygen saturation, *V–V* veno-venous

Intestinal tissue oxygen saturation (SO_2_), as assessed by white light and laser Doppler spectrometry, was impaired during low-flow (60 mL/kg/min; V–V 60; 65% [62%, 70%], *p* = 0.003) but not high-flow (90 mL/kg/min; V–V 90; 74% [71%, 80%], *p* = 1.000) V–V ECMO therapy compared to sham therapy (76% [72%, 79%]; Fig. 1A). In addition, hepatic SO_2_ and relative haemoglobin (Hb) were reduced during V–V 60 (21% [14%, 26%] and 67 [62, 70] relative Units (RU)) and V–V 90 (19% [16%, 21%] and 73 [59, 82] RU) ECMO therapy compared to sham therapy (43% [38%, 48%] and 77 [72, 80] RU; all *p* ≤ 0.003; Fig. 1D, F). However, intestinal and hepatic blood flow did not differ significantly between the V–V 60 (319 [309,353] RU and 198 [179,216] RU), V–V 90 (339 [311,371] RU and 213 [197,229] RU), and sham (345 [302,362] RU and 193 [185,213] RU) therapies (Fig. 1B, E).

### Hemodynamic parameters

**Fig. 2:**
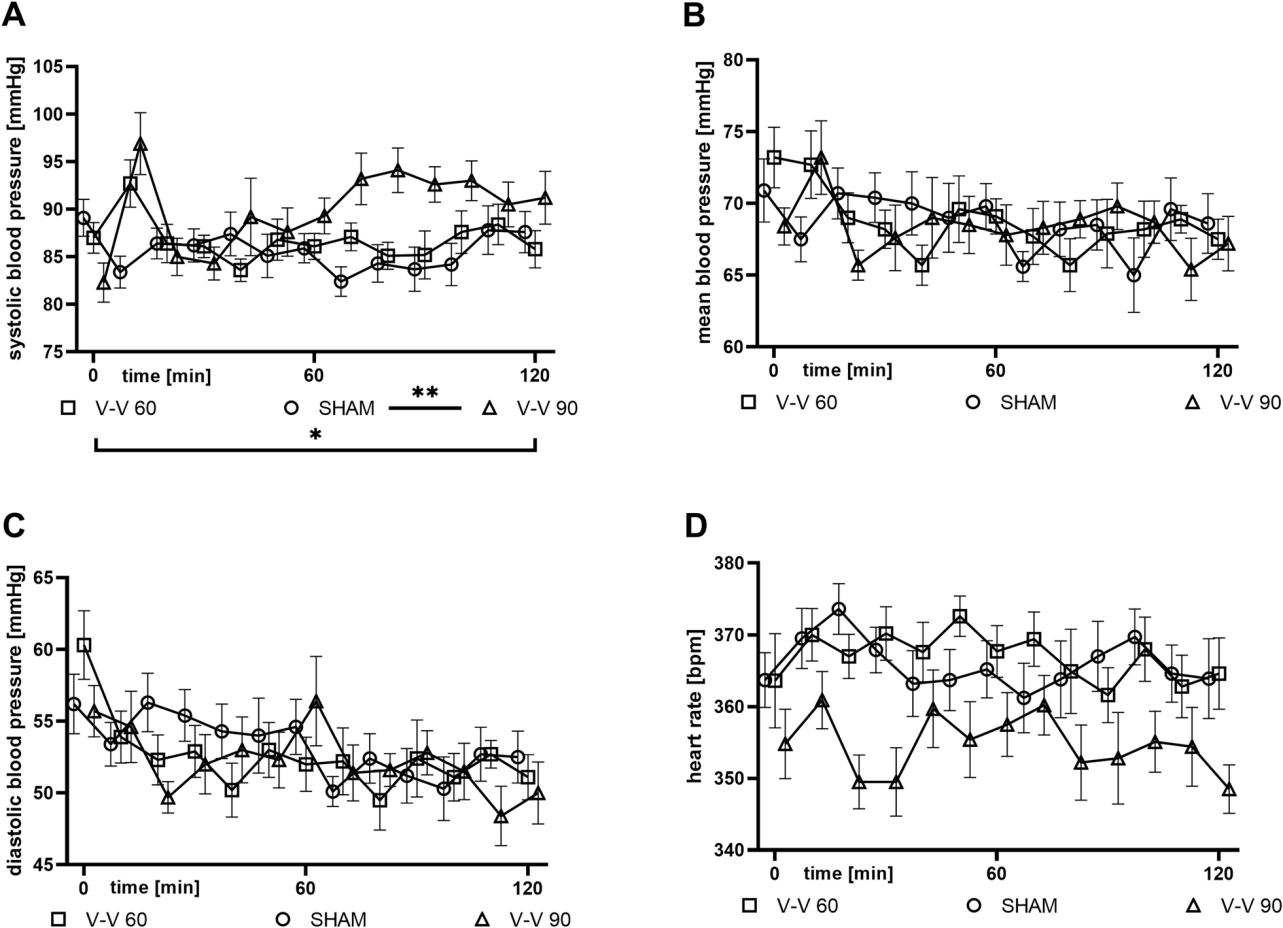
Time course of **A** SAP, **B** MAP, **C** DAP, and **D** heart rate. SAP was higher during V–V 90 ECMO therapy than V–V 60 ECMO therapy and sham therapy. MAP, DAP, and heart rate did not differ significantly between therapies (*n* = 10/group). The asterisks denote the degree of statistical significance: *, *p* < 0.05; **, *p* < 0.01. *DAP* diastolic arterial pressure, *ECMO* extracorporeal membrane oxygenation, *MAP *mean arterial pressure, *SAP* systolic arterial pressure, *V–V* veno-venous

Systolic arterial pressures (SAP) were higher during V–V 90 (*p* = 0.003) and V–V 60 (*p* = 0.035) ECMO therapy than sham therapy (Fig. 2). However, mean (MAP) and diastolic (DAP) arterial blood pressure and heart rate did not differ significantly between the V–V ECMO and sham therapies (Fig. 2).

**Fig. 3:**
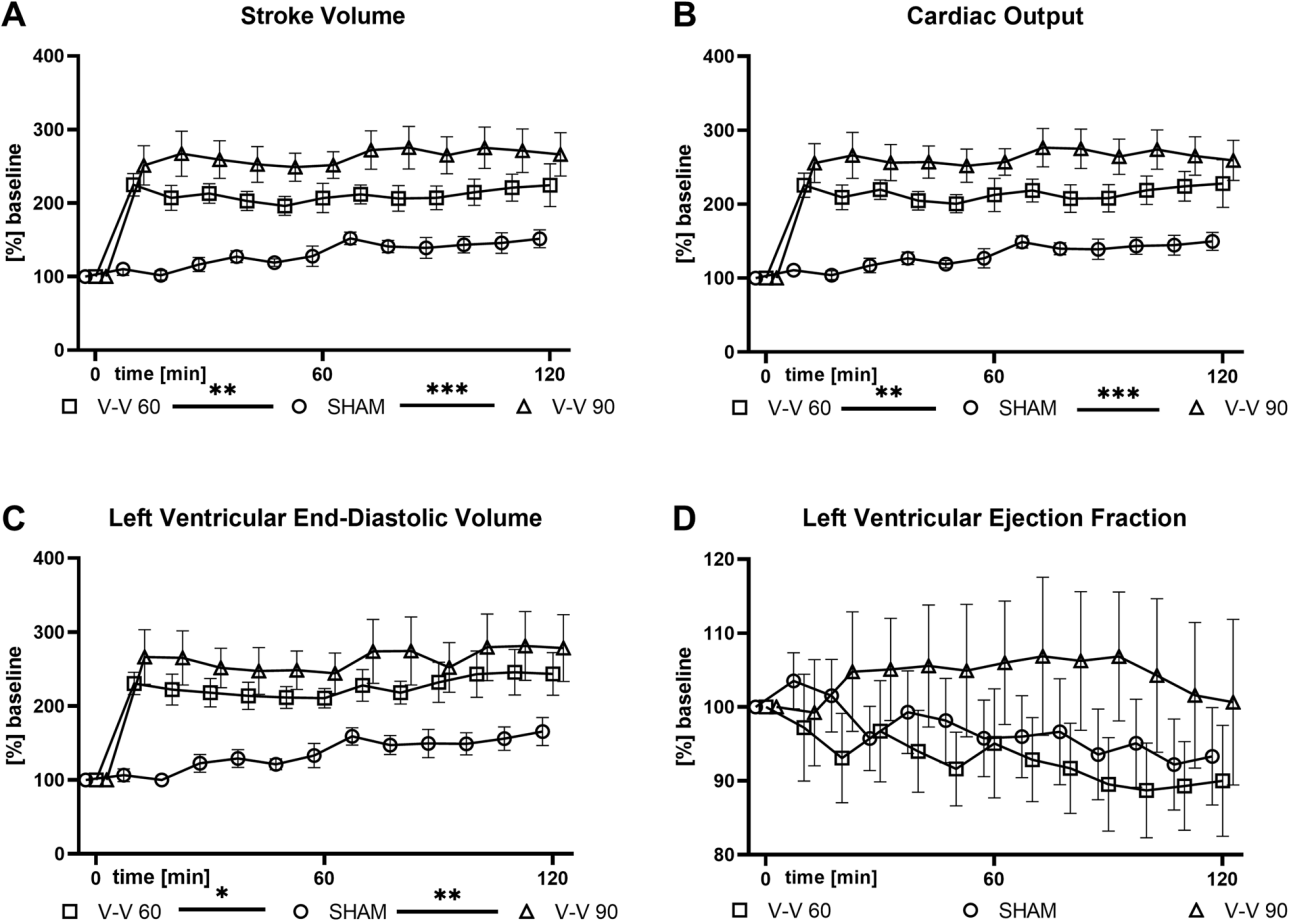
Time course of **A** SV, **B** CO, **C** LVEDV, and **D** LVEF. SV, CO, and LVEDV were significantly higher during both V–V 60 and 90 ECMO therapy than sham therapy. LVEF did not differ significantly between (*n* = 10/group). The asterisks denote the degree of statistical significance: *, *p* < 0.05; **, *p* < 0.01; ***, *p* < 0.001. *CO*  cardiac output, *ECMO*  extracorporeal membrane oxygenation, *LVEDV * left ventricular end-diastolic volume, *LVEF * left ventricular ejection fraction, *SV*  stroke volume, *V–V*  veno-venous

Analysis of the conductance catheter data revealed elevated stroke volume (SV), cardiac output (CO), and left ventricular end-diastolic volume (LVEDV) during V–V 60 (*p* = 0.004, *p* = 0.002, and *p* = 0.038) and V–V 90 (*p* < 0.001, *p* < 0.001, and *p* = 0.003) ECMO therapy compared to sham therapy (Fig. 3). However, left ventricular ejection fraction (LVEF, Fig. 3D) and left ventricular end-diastolic pressure (LVEDP, data not shown) did not differ significantly between the V–V ECMO and sham therapies.

### Blood gas analysis

While arterial oxygen saturation (S_a_O_2_) did not differ significantly between the V–V ECMO and sham therapies, central venous oxygen saturation (S_cv_O_2_) was significantly lower during V–V 60 ECMO therapy than sham therapy (*p* = 0.042, Table 1). The arterial partial pressure of carbon dioxide (pCO_2_) was also significantly lower during V–V 90 ECMO therapy than sham therapy (*p* = 0.001, Table 1). Moreover, Hb levels and haematocrit (Hct) were significantly lower during V–V 60 and V–V 90 ECMO therapy than sham therapy (all *p* < 0.001). While lactate (Lac) and sodium (Na) levels were significantly elevated during V–V 60 (*p* = 0.025 and *p* = 0.019) and V–V 90 (*p* = 0.003 and *p* < 0.001) ECMO therapy compared to sham therapy, glucose (Glu) and potassium (K) levels were only significantly lower during V–V 90 ECMO therapy (*p* = 0.002 and *p* = 0.007). Furthermore, chloride (Cl) levels were only elevated during V–V 90 therapy compared to sham therapy (*p* < 0.001). However, calcium (Ca), base excess (BE), pH, and arterial partial pressure of oxygen (pO_2_) did not differ significantly between the V–V ECMO and sham therapies (Table 1).

**Table 1:** Results of the blood gas analysis

		0 min	30 min	60 min	90 min	120 min
S_a_O_2_ [%]	Sham	100 [100,100]	100 [100,100]	100 [100,100]	100 [99,100]	100 [99,100]
	V–V 60	100 [100,100]	100 [98,100]	100 [97,100]	100 [96,100]	98 [96,100]
	V–V 90	100 [100,100]	100 [100,100]	100 [100,100]	100 [100,100]	100 [99,100]
S_cv_O_2_ [%]	Sham	75 [68,77]	78 [74,82]	79 [75,84]	77 [77,80]	75 [73,82]
*	V–V 60	72 [67,73]	77 [72,82]	73 [66,76]	69 [67,76]	62 [59,67]
	V–V 90	71 [68,74]	82 [78,84]	74 [72,78]	73 [67,78]	68 [62,77]
pO_2_ [mmHg]	sham	141 [124,156]	128 [127,140]	130 [116,132]	121 [107,124]	114 [105,125]
	V–V 60	148 [138,157]	125 [91,149]	105 [88,134]	97 [82,116]	86 [78,108]
	V–V 90	152 [142,164]	139 [121,162]	119 [114,147]	113 [97,126]	112 [94,118]
pCO_2_ [mmHg]	sham	42 [40,44]	43 [42,45]	42 [38,44]	43 [40,44]	42 [41,44]
	V–V 60	41 [39,46]	40 [36,44]	39 [37,41]	40 [39,42]	42 [40,44]
**	V–V 90	38 [34,42]	37 [35,41]	39 [37,41]	38 [36,39]	38 [36,40]
pH	sham	7.39 [7.36,7.41]	7.39 [7.36,7.42]	7.39 [7.35,7.41]	7.36 [7.35,7.41]	7.36 [7.35,7.39]
	V–V 60	7.38 [7.33,7.40]	7.43 [7.37,7.45]	7.41 [7.38,7.45]	7.40 [7.38,7.42]	7.39 [7.37,7.40]
	V–V 90	7.38 [7.37,7.43]	7.39 [7.35,7.43]	7.40 [7.39,7.41]	7.39 [7.37,7.42]	7.40 [7.37,7.42]
BE	sham	0.4 [− 1.8,0.9]	− 0.2 [− 1.2, − 1.6]	− 0.6 [− 1.8,0.4]	− 1.2 [− 1.8,0.7]	− 0.9 [− 1.3, − 0.3]
	V–V 60	− 1.2 [− 2.2,0.1]	1.2 [0.2,2.0]	0.8 [− 0.7,1.9]	0.1 [− 0.8,1.4]	0.6 [− 0.8,1.9]
	V–V 90	− 1.6 [− 2.6, − 1.0]	− 1.7 [− 3.0, − 1.1]	− 1.0 [− 1.6,0.2]	− 1.8 [− 2.7, − 0.3]	− 1.1 [− 1.6, − 0.6]
Lac [mmol/L]	sham	1.3 [1.1,1.8]	0.8 [0.6,1.0]	0.8 [0.7,0.9]	1.0 [0.9,1.1]	0.9 [0.9,1.1]
*	V–V 60	1.4 [0.9,1.7]	1.1 [1.0,1.4]	0.8 [0.7,1.1]	1.2 [1.1,1.2]	1.2 [1.1,1.3]
**	V–V 90	1.5 [1.0,1.6]	1.2 [1.1,1.4]	1.0 [0.9,1.2]	1.2 [1.1,1.5]	1.2 [1.0,1.3]
Hb [mg/dL]	sham	13.8 [12.5,14.3]	12.2 [11.4,13.1]	12.0 [10.8,12.6]	10.9 [9.6,11.8]	10.8 [9.1,11.5]
***	V–V 60	14.1 [13.6,14.3]	7.8 [7.5,8.0]	7.6 [7.3,8.1]	7.1 [6.9,7.6]	7.3 [6.6,7.8]
***	V–V 90	14.4 [14.1,14.8]	7.6 [7.4,7.8]	7.5 [7.3,8.0]	7.4 [6.8,7.6]	7.1 [6.8,7.5]
Hct [%]	sham	42.3 [38.4,43.7]	37.5 [35.1,40.4]	36.9 [33.2,38.5]	33.7 [29.6,36.5]	33.4 [28.4,35.3]
***	V–V 60	43.3 [41.9,44.0]	24.2 [23.4,25.0]	23.7 [22.7,25.2]	22.1 [21.4,23.7]	22.6 [20.6,24.3]
***	V–V 90	44.2 [43.0,45.4]	23.7 [22.9,24.2]	23.4 [22.8,25.0]	22.9 [21.4,23.8]	22.1 [21.1,23.2]
Glu [mg/dL]	sham	166 [161,178]	142 [132,149]	129 [124,134]	127 [124,131]	126 [117,137]
	V–V 60	175 [156,185]	139 [120,151]	124 [120,131]	120 [116,129]	121 [117,129]
**	V–V 90	161 [140,171]	126 [121,131]	117 [112,122]	114 [111,117]	118 [111,124]
Na [mmol/L]	sham	141 [141,143]	141 [141,143]	142 [141,143]	142 [142,144]	143 [142,144]
*	V–V 60	143 [142,143]	143 [142,144]	143 [143,145]	145 [143,145]	144 [143,145]
***	V–V 90	144 [142,144]	144 [143,145]	145 [144,146]	146 [144,146]	145 [144,146]
K [mmol/L]	sham	4.0 [3.9,4.1]	4.4 [4.3,4.5]	4.2 [4.1,4.4]	4.2 [4.2,4.4]	4.3 [4.1,4.4]
	V–V 60	4.2 [4.0,4.3]	4.0 [4.0,4.2]	4.1 [3.9,4.2]	4.1 [4.0,4.1]	4.2 [4.1,4.3]
**	V–V 90	4.1 [3.9,4.3]	4.0 [3.8,4.1]	4.1 [3.9,4.1]	4.1 [3.9,4.3]	4.1 [4.0,4.2]
Cl [mmol/L]	sham	108 [107,109]	109 [107,111]	110 [108,111]	111 [110,111]	111 [110,113]
	V–V 60	108 [107,108]	110 [109,110]	111 [110,111]	111 [111,112]	112 [111,112]
***	V–V 90	109 [108,110]	112 [110,113]	112 [111,113]	113 [113,114]	113 [112,114]
Ca [mmol/L]	sham	1.49 [1.47,1.53]	1.49 [1.47,1.53]	1.50 [1.49,1.53]	1.49 [1.49,1.53]	1.50 [1.48,1.51]
	V–V 60	1.51 [1.49,1.53]	1.51 [1.46,1.52]	1.51 [1.47,1.53]	1.50 [1.48,1.51]	1.49 [1.47,1.51]
	V–V 90	1.50 [1.49,1.53]	1.54 [1.51,1.56]	1.53 [1.52,1.54]	1.53 [1.50,1.55]	1.52 [1.49,1.53]

The data are presented as the median (25th, 75th percentiles). The degree of statistical significance of the differences between the sham, V–V 60, and V–V 90 ECMO therapies is denoted by asterisks: *, *p* < 0.05; **, *p* < 0.01; ***, *p* < 0.001. *BE* base excess, *Ca* calcium, *Cl* chloride, *Glu* glucose, *Hb* haemoglobin, *Hct* haematocrit, *K* potassium, *Lac* lactate, *Na* sodium, *pCO*_*2*_ arterial partial pressure of carbon dioxide, *pO*_*2*_ arterial partial pressure of oxygen, *S*_*a*_*O*_*2*_ arterial oxygen saturation, *S*_*cv*_*O*_*2*_ central venous oxygen saturation, *V–V* veno-venous

### Inflammatory parameters

**Fig. 4:**
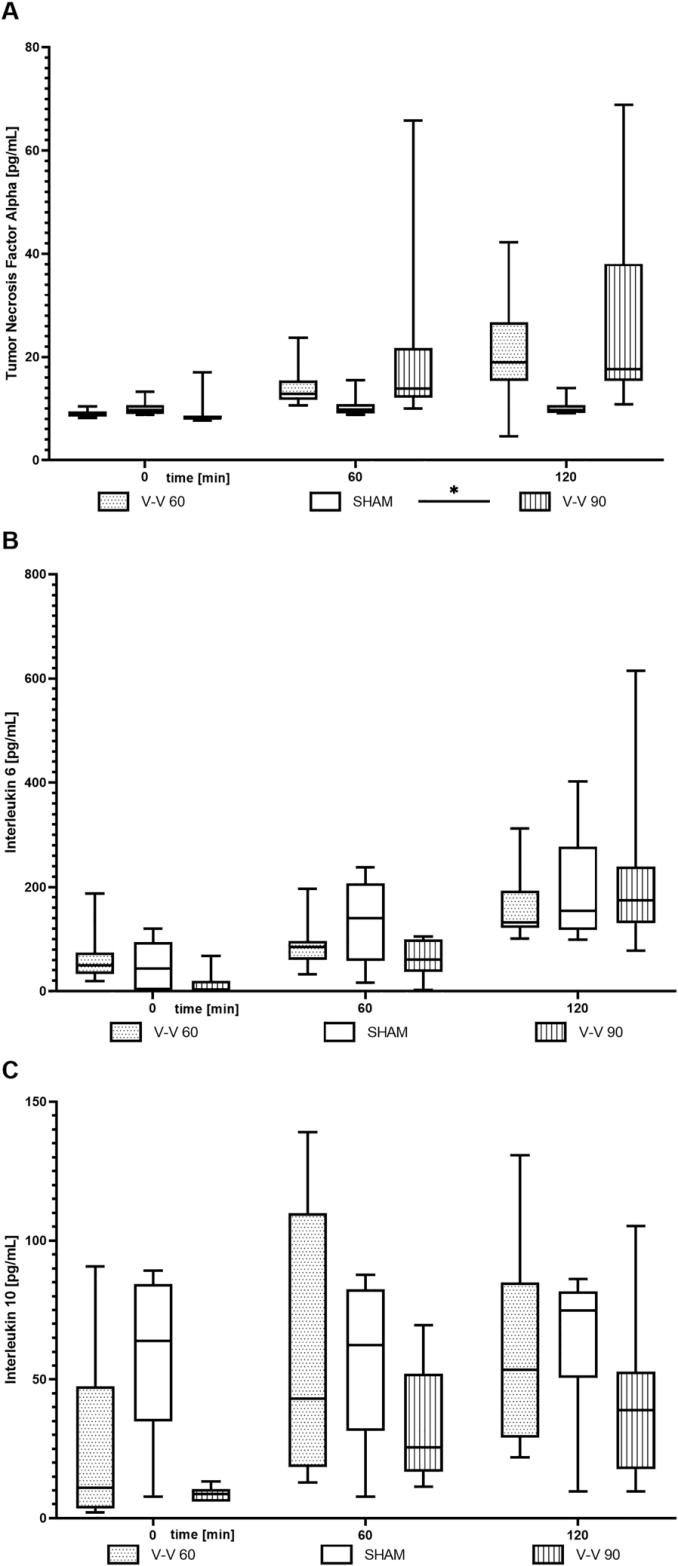
Time course of the plasma levels of the inflammatory markers **A** TNF-α, **B** IL6, and **C** IL10. While TNF-α levels were significantly higher during V–V 90 ECMO therapy than sham therapy, IL6 and IL10 levels did not differ significantly between the V–V ECMO and sham therapies (*n* = 10/group). The asterisks denote the degree of statistical significance: *, *p* < 0.05. Box and whisker plots show the median, interquartile range (box), and minimum and maximum (whiskers). *IL6* interleukin 6, *IL10* interleukin 10, *TNF-α* tumour necrosis factor-alpha

While tumour necrosis factor-alpha (TNF-α) levels were elevated only during V–V 90 ECMO therapy compared to sham therapy (*p* = 0.033), interleukin 6 (IL6) or interleukin 10 (IL10) levels did not differ significantly between the V–V ECMO and sham therapies (Fig. 4). However, the levels of C–X–C motif ligands 2 (CXCL2) and 5 (CXCL5) were below the detection limit of the enzyme-linked immunosorbent assays (ELISAs).

**Fig. 5:**
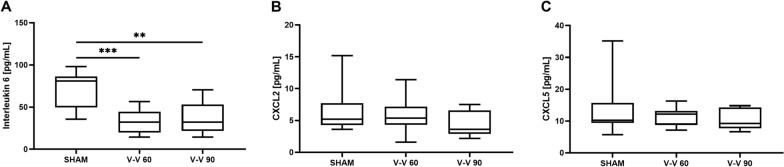
BAL levels of the inflammatory markers **A** IL6, **B** CXCL2, and **C** CXCL5. While IL6 levels were significantly lower during both V–V 60 and V–V 90 ECMO therapies than sham therapy, CXCL2 and CXCL5 levels did not differ significantly between the V–V ECMO and sham therapies (*n* = 10/group). The asterisks denote the degree of statistical significance: ***, *p* < 0.001; **, *p* < 0.01. Box and whisker plots show the median, interquartile range (box), and minimum and maximum (whiskers). *BAL* broncho alveolar lavage, *CXCL2 *C–X–C motif ligand 2, *CXCL5* C–X–C motif ligand 5, *IL6* interleukin 6, *IL10* interleukin 10, *V–V* veno-venous

In addition, bronchoalveolar lavage (BAL) IL6 levels were lower during the V–V 60 and V–V 90 ECMO therapies than the sham therapy (*p* < 0.001 and *p* = 0.001, Fig. 5A). However, BAL levels of CXCL2 and CXCL5 did not differ significantly between the V–V ECMO and sham therapies (Fig. 5B, C). Notably, the BAL levels of TNF-α and IL10 were below the detection limit of the ELISAs.

## Discussion

To the best of our knowledge, this study is the first to examine the intestinal and hepatic microcirculation in vivo during V–V ECMO therapy with femoral drainage and jugular return in rats without septic shock. It revealed three main findings. First, it demonstrated reduced intestinal SO_2_ only during V–V 60 ECMO therapy and impaired hepatic SO_2_ and relative Hb concentrations during both V–V 60 and V–V 90 ECMO therapies. Second, while SV, CO, and LVEDV were elevated during both V–V 60 and V–V 90 ECMO therapy, SAP was only elevated during V–V 90 ECMO therapy. Third, while TNF-α levels were only elevated during V–V 90 ECMO therapy, BAL levels of IL6 were lower during both V–V 60 and V–V 90 ECMO therapy.

While measuring the intestinal microcirculation during V–V ECMO therapy is challenging in critically ill patients and patients developing abdominal complications, our results are of great interest [14]. We previously established measuring intestinal and hepatic microcirculation using micro-light guide spectrophotometry in a rat model of septic shock during V–V ECMO therapy [13]. Reduced intestinal SO_2_ and relative Hb concentrations were seen during septic shock. In contrast, an impaired intestinal SO_2_ was only recognised during V–V 60 ECMO therapy in this study. However, the inspiratory oxygen fraction on the membrane was adjusted to 0.5 (as it was set in our previous study) to gain comparable results. Therefore, the combination of low blood flow and lung protective ventilation might explain the lower intestinal SO_2_. Unlike in this study, only high blood flow was used in our previous study. Notably, that study observed no differences in intestinal SO_2_ between groups during high-flow ECMO therapy. Therefore, the reason for the reduced intestinal microcirculation in rats with septic shock during V–V ECMO therapy seems to be the combination of septic shock with V–V ECMO therapy in the context of a second hit model. Further studies are needed to clarify if these changes are also present in ARDS.

Given the lack of comparable studies examining V–V ECMO therapy in humans, we compare our results to those in humans during cardiopulmonary bypass (CPB). Thorén et al. measured jejunal mucosal perfusion in humans using laser Doppler flowmetry during CPB, observing an increase in jejunal mucosal perfusion during mild hypothermic CPB [15]. However, it must be underscored that the V–V ECMO machine cannot be directly compared with the heart–lung machine used during CPB. Besides the surgical impact on systemic inflammation, the effects of the suction pumps and the difference in the cannulation strategy cannot be ignored.

Interestingly, the results of the hepatic microcirculation are similar to our findings during septic shock and V–V ECMO therapy. Both the hepatic SO_2_ and relative Hb concentrations were reduced independent of the blood flow. However, the effect of the draining cannula on the hepatic SO_2_ remains unclear. Hepatic stasis should be avoided due to the multi-orifice design of the draining cannula. Because the results can be seen independent of septic shock, a potential influence of the lipopolysaccharide on hepatic microcirculation seems unreasonable. However, the hepatic probe is designed with an adhesive surface, which might offer an explanation for the reduced hepatic SO_2_.

Next, the hemodynamic data, reflecting the macrocirculation were analysed to identify potential explanations for the reduced hepatic and intestinal microcirculation during V–V ECMO therapy. While systolic blood pressure was only elevated during V–V 90 ECMO therapy in this study, these results are consistent with our previous studies using healthy rats undergoing femoral–jugular V–V ECMO therapy [10]. Contrary to our results, Fujii et al. reported a reduced MAP during low flow (50–60 mL/kg/min) V–V ECMO therapy in rats [16]. However, they drained blood via cannulation of the right atrium. Since the blood was returned to a femoral vein, recirculation cannot be excluded in their study design, which could explain the reduced MAP.

Interestingly, in this study, SV and CO were elevated during both V–V 60 and V–V 90 ECMO therapy. Again, these results are comparable to our previous study involving rats undergoing V–V ECMO therapy [11]. The return of oxygenated blood, which is being pumped through the lungs, could have reduced pulmonary vascular resistance, since oxygen is known to be a potent pulmonary vasodilator. The increased left ventricle preload might also have resulted in increased SV and, thus, CO. However, no increase in S_cv_O_2_ was observed during our experiments. It should be noted that ScvO₂ was sampled from the venous reservoir of the ECMO circuit (or the draining cannula in sham animals) and, therefore, reflects venous oxygen saturation of the lower body rather than the true mixed venous oxygen saturation entering the lungs. Measuring mixed venous oxygen saturation or pulmonary artery resistance in rats is technically very challenging. Nevertheless, the reduction in arterial pCO₂ observed during high ECMO blood flow is known to decrease pulmonary vascular resistance. In addition, all rats developed acute dilutional anaemia, and normovolemic anaemia is associated with a compensatory increase in cardiac output [16]. This mechanism may provide an additional explanation for the elevated CO observed in this study.

Despite the increased SV and CO, intestinal and hepatic SO_2_ were reduced in this study. Therefore, we next examined the blood gas data. The pCO_2_ was lower during V–V 90 ECMO therapy than the sham therapy. Since the blood gases are evaluated only every 30 min, adjusting the sweep gas flow on the ECMO membrane remains challenging. Nonetheless, it has to be underscored that the reported values were within the intended range.

Furthermore, dilutional anaemia was observed during V–V ECMO therapy. However, the measured values were consistent with our previous studies. While significantly elevated Lac levels were seen during V–V ECMO therapy compared to sham therapy, no values > 2 mmol/L were observed. Therefore, the critical Hb concentration did not appear to be achieved. Since studies have demonstrated that reduced Hb concentrations are associated with reduced oxygenation, as measured by near-infrared spectroscopy, the reduced intestinal SO_2_ in this study could be caused by dilutional anaemia [17]. In addition, no differences in Hb concentration were seen between the V–V 60 and V–V 90 ECMO therapies, and intestinal SO_2_ was only reduced during V–V 60 ECMO therapy. However, S_cv_O_2_ was lower during V–V 60 ECMO therapy than sham therapy. Since lung-protective ventilation with reduced tidal volume and respiratory rate was used in both V–V 60 and V–V 90 ECMO therapies, oxygen delivery might be lower during the V–V 60 therapy, resulting in increased oxygen extraction. This assumption might also explain the reduced intestinal SO_2_, which was only observed during V–V 60 ECMO therapy.

The priming of the ECMO circuit using unbalanced hydroxyethyl starch containing 154 mmol/L of Na and Cl may explain the measured changes in the electrolytes.

This study verified the ECMO-induced inflammation, which was reflected by increased serum TNF-α levels. These results are consistent with our previous study [11]. It should be emphasized that ECMO-induced haemodilution influences cytokine concentrations throughout the experiment, which may partly account for the relatively small differences observed between groups. Notably, after 2 h, the median TNF-α concentration in both ECMO groups exceeded the maximum value recorded in the sham group. McILwain et al. reported in piglets undergoing V–A ECMO that TNF-α levels rose significantly after 1 h, whereas increases in IL-6 were not observed until 4 h of therapy [18]. This temporal pattern may explain the absence of IL-6 differences in this study within the 2-h timeframe. Interestingly, serum cytokine levels were not significantly elevated during V–V 60 ECMO. This suggests that the inflammatory response could be influenced by blood flow through the pump and membrane. However, as significant between-group differences for TNF-α emerged only after 2 h of ECMO therapy, this interpretation should be regarded with caution.

Notably, lung-protective ventilation with reduced tidal volume and respiratory rate was applied during V–V ECMO therapy. It is well-established that elevated driving pressures and tidal volumes are associated with epithelial overstretching and, consequently, ventilation-associated lung injury [19]. Therefore, the reduced driving pressures and tidal volumes during lung-protective ventilation in this study may explain the decreased BAL levels of the pro-inflammatory marker IL-6 during V–V ECMO therapy. Interestingly, these results contrast with our previous study involving rats with septic shock undergoing V–V ECMO therapy, where it was associated with increased levels of proinflammatory markers in the BAL [13]. In both studies, rats received lung-protective ventilation. Based on our results, it seems that pulmonary inflammation was triggered by a second hit, like the septic shock induced by lipopolysaccharide.

This study had some limitations that must be acknowledged. First, while the mucosal microcirculation should be measured from inside the intestine, the micro-light guide spectrophotometry probes were placed on the outside. Nonetheless, the probes are designed to measure oxygenation at a depth of 2–4 mm, so their results can be used as a surrogate for intestinal microcirculation. Nevertheless, the results do not reflect global perfusion. Second, the hepatic probe was placed on the liver lobe, so a local compression cannot be excluded. Nonetheless, significant differences in hepatic microcirculation were observed between V–V ECMO and sham therapies. Third, recirculation between the draining and returning cannula was not directly measured in this study. However, the correct position of the draining cannula in the inferior vena cava was verified in our own previous work [11]. Since venous recirculation would be expected to result in a reduced preload and subsequently lower cardiac output of the left heart, the observation of increased stroke volume and cardiac output in this study suggests that relevant recirculation is unlikely. Fourth, since the animals in the ECMO groups were ventilated in a lung-protective manner, the results on pulmonary inflammation cannot be directly compared. An additional group with lung-protective ventilation and without extracorporeal membrane oxygenation would, however, likely have been associated with hypercapnia and hypoxia. Finally, the results of healthy animal studies cannot be directly transferred to critically ill humans. While the heart rate is faster, the cardiopulmonary system of rats is similar to humans [20]. Nevertheless, further studies are needed to investigate the molecular mechanisms underlying the different pulmonary responses to ECMO therapy.

## Conclusions

Contrary to the results in rats with septic shock, no difference in intestinal microcirculation was seen between V–V 90 ECMO and sham therapy in healthy rats. Therefore, combining septic shock with ECMO-induced inflammation seems to be the cause of the reduced intestinal microcirculation in rats with septic shock during V–V ECMO therapy. In addition, reduced hepatic microcirculation was seen independent of blood flow. Furthermore, ECMO-related cytokine changes showed a flow-dependent trend. Compared to rats with septic shock, pulmonary inflammation was lower in healthy rats during lung-protective ventilation and V–V ECMO therapy independent of blood flow. Hypothetically, pulmonary inflammation may be influenced by a second hit, such as septic shock or acute respiratory distress syndrome.

## Methods

### Animals

All procedures involving animals were conducted in compliance with standards for animal care and approved by the Animal Welfare Commission of the Department of Veterinary Medicine at the Regional Council Giessen (GI 20/26 Nr. G 77/2019; Regierungspraesidium Giessen, Germany). This study was reported according to the Animal Research: Reporting of In Vivo Experiments guidelines [21].

Male Lewis rats (330–350 g) were obtained from Janvier Labs (Le Genest St. Isle, France) and kept at 22 °C, 55% relative humidity, and a 14/10-h day/night cycle, with ad libitum access to standard chow and water. The rats were randomly divided into three groups per lot to receive V–V 60, V–V 90, or sham therapy (*n* = 10/group). During sham therapy, all cannulas were placed, and the rats were observed for 2 h without V–V ECMO.

### Induction and maintenance of anaesthesia

The rats were intubated orotracheally (16 G cannula; B. Braun, Melsungen, Germany) after inhalation induction of anaesthesia with 5% isoflurane (Baxter, Unterschleißheim, Germany) balanced with 95% oxygen and ventilated with an inspiratory oxygen fraction of 0.5 in a volume-controlled and weight-adjusted manner (tidal volume = 6.2 mL × body weight [kg]^1.01^, respiratory rate = 53.3 × body weight [kg]^−0.26^) using a Harvard Inspira ventilator (Harvard Apparatus, Cambridge, UK). The rats were fixed on a heating pad, which was controlled using the temperature measured by a rectal probe. Additional heat was provided with an infrared lamp to adjust the rectal temperature between 36.5 °C and 37 °C.

Next, the electrocardiograph was connected, and the lateral tail vein was punctured percutaneously for the continuous infusion of fentanyl (10 µg/kg/h; Albrecht GmbH, Aulendorf, Germany), midazolam (2 mg/kg/h; Roche, Basel, Switzerland), pancuronium (0.1 mg/kg/h; Inresa, Freiburg, Germany), and a balanced crystalloid solution (5 mL/kg Sterofundin; B. Braun, Melsungen, Germany) [11, 13, 22, 23].

### Cannulation and abdominal laparotomy

The following vascular cannulas were placed after surgical preparation as previously described [11, 13]. First, the tail artery was punctured with a 24 G cannula for continuous measurement of the arterial blood pressure and intermittent blood gas analysis (B. Braun, Melsungen, Germany). After the neck was opened with a small skin incision, the right carotid artery and internal jugular vein were dissected. Next, a 2F pressure–volume catheter (SPR-838; Millar, Houston, TX, USA) was carefully inserted through the carotid artery into the left ventricle to measure the SV, CO, LVEDV, LVEDP, and LVEF. Then, a shortened modified 20 G cannula (Surflo, Terumo, Eschborn, Germany) was placed in the internal jugular vein for the ECMO return and connected to a three-way stopcock.

After a median skin incision was made with scissors, the abdominal cavity was opened via electrocautery (AA01 Bovie high-temperature cautery; Bovie Medical Corporation, Clearwater, FL, USA). Next, the intestine was mobilised, and the vascular-free mesentery of a small intestine loop was dissected. Then, the probe for the white light and laser Doppler spectrometry (LFX-151; LEA Medizintechnik GmbH, Heuchelheim, Germany) was placed on the intestine and secured with a one-side-open silicon tube (8 mm diameter) to assure a loose fit. Next, the whole intestine was put back in its original position. Then, a shallow well was placed on the right lobe of the liver (LFX-45; LEA Medizintechnik GmbH, Heuchelheim, Germany), and the abdominal cavity was covered with a warm, wet compress [13, 24, 25].

A small skin incision was made in the groin, and the right femoral vein was dissected. Finally, all rats received heparin (400 IU/kg; Merckle GmbH, Blaubeuren, Germany), and the femoral vein was cannulated with a custom-modified multi-orifice 18 G cannula (Surflo; Terumo, Eschborn, Germany) for venous drainage to the ECMO circuit, as previously described in detail [11], and connected to a three-way stopcock.

### Extracorporeal membrane oxygenation

As previously described, the ECMO circuit consisted of a roller pump (Verderflex Vantage 3000; Verder Ltd, Castleford, UK), a venous reservoir (M. Humbs, Valley, Germany), and a membrane oxygenator (Micro-1; Kewei Rising Medical, Shenzhen, China) [11, 13, 22–24]. A Heidelberger extension line (B. Braun, Melsungen, Germany) was wrapped around the oxygenator and connected to a heating pump (HU35; Gettinge, Raststatt, Germany) to prevent heat loss. The draining cannula was connected with a shortened Heidelberg extension line (B. Braun, Melsungen, Germany) to the venous reservoir, and the membrane oxygenator was linked to the return cannula by a shortened syringe pump line (B. Braun, Melsungen, Germany). A three-way stopcock (B. Braun, Melsungen, Germany) was connected to the venous reservoir for central venous blood gas analysis. The whole circuit was primed with 250 IU of heparin (Ratiopharm, Ulm, Germany) and 9 mL of 6% unbalanced hydroxyl ethyl starch (Voluven; Fresenius Kabi, Bad Homburg, Germany). The blood flow was started at 30 mL/kg/min and then continuously increased to the target flow of 60 (V–V 60) or 90 (V–V 90) mL/kg/min. The sweep gas flow on the membrane was adjusted between 20 and 70 mL/min to regulate the pCO_2_ between 35 and 45 mmHg. The oxygen fraction on the ECMO membrane was set to 0.5. After reaching the target blood flow, lung-protective ventilation was achieved by adjusting the respiratory rate and tidal volume at 75% of the rat’s weight according to the formulae:

$${\text{tidal volume}}\,\, = \,\,{6}.{\text{2 mL}}\, \times \,{\text{body weight }}\left( {{\text{kg}}} \right)^{{{1}.0{1}}} {\text{and respiratory rate}}\,\, = \,\,{53}.{3}\, \times \,{\text{body weight }}\left( {{\text{kg}}} \right)^{{ - 0.{26}}}$$

### Intestinal microcirculation

To access the intestinal and hepatic microcirculation, the intestine and liver probes were connected to the micro-light guide spectrophotometer (LEA Medizintechnik GmbH, Heuchelheim, Germany). Each probe consisted of two light sources and their corresponding optical sensors, as previously described [13, 24, 25]. White light spectroscopy (450–1000 nm) was used to measure the percentage of SO_2_, which is composed primarily of venous and secondarily of arterial and capillary oxygen saturation. The amount of light absorption due to Hb was analysed and expressed in RU. The second light source emitted laser light (820 nm, 30 mW) and was used to determine erythrocyte velocity and, thus, relative blood flow, which was expressed in RU [13, 24, 25].

### Timepoints of hemodynamic measurements

Baseline values were recorded before V–V ECMO began, and subsequent measurements were captured every 10 min for up to 120 min.

### Blood analyses

Blood gas analyses were performed immediately before starting ECMO and then every 30 min for up to 120 min (ABL800; Radiometer, Copenhagen, Denmark). Recordings consisted of S_a_O_2_, S_cv_O_2_, pO_2_, pCO_2_, Hb, Hct, pH, BE, Lac, Glu, Na, K, Ca, and Cl. Blood samples were taken for inflammation analysis immediately after commencing ECMO and then every 60 min for up to 120 min. This blood was immediately centrifuged at 5000 rpm and 4 °C for 5 min, and the plasma samples were stored at − 80 °C until needed.

### End of the experiments

After 120 min, isoflurane was adjusted to 5%, and the rats were euthanised by exsanguination through the draining ECMO cannula. Immediately after confirming death by asystole, the neck was opened, and the trachea was dissected. Next, a ligature was fixed around the trachea to seal the tube. Then, the lungs were flushed repeatedly with 40 mL of balanced crystalloid solution (Sterofundin; Fresenius, Bad Homburg, Germany). After centrifuging the BAL at 1200 rpm and 4 °C for 8 min, the supernatant was collected and stored at − 80 °C until needed [13, 24].

### ELISAs

Systemic and pulmonary inflammation was assessed based on the concentrations of the cytokines TNF-α, IL6, IL10, CXCL2, and CXCL5 in the plasma and BAL measured using commercial ELISA kits (R6000B, RTA00, and R1000 from R&D System [Wiesbaden, Germany] and ERCXCL2 and ERCXCL5 from Thermo Fisher Scientific [Waltham, MA, USA]) according to the manufacturer’s instructions. The probes were only thawed once.

### Statistical analyses

All data are expressed as the median (25th and 75th percentiles). Groups were compared using repeated measures analysis of variance followed by post-hoc Bonferroni tests. Because the baseline values were measured without ECMO support, they were excluded from the analysis. An inbred rat strain was used to reduce the variance between groups. Considering alpha and beta error rates of 0.05 and 0.02, respectively, groups with 10 animals resulted in an effect size of 0.55 (moderate), as calculated using G*Power (version 3.1.9.2, Heinrich Heine University, Duesseldorf, Germany). A *p* value of < 0.05 was considered statistically significant. All statistical analyses were performed using SPSS (version 20; IBM, Stuttgart, Germany). All graphs were created using GraphPad Prism (version 7; GraphPad Software, San Diego, CA, USA).

## Data Availability

The data sets used and/or analysed during the current study are available from the corresponding author on reasonable request.

## Abbreviations

BAL: Bronchoalveolar lavage
BE: Base excess
Ca: Calcium
Cl: Chloride
CO: Cardiac output
CPB: Cardiopulmonary bypass
CXCL2: C–X–C motif ligand 2
CXCL5: C–X–C motif ligand 5
DAP: Diastolic arterial pressure
ECMO: Extracorporeal membrane oxygenation
ELISA: Enzyme-linked immunosorbent assay
Glu: Glucose
Hb: Haemoglobin
Hct: Haematocrit
IL10: Interleukin 10
IL6: Interleukin 6
K: Potassium
Lac: Lactate
LVEDP: Left ventricular end-diastolic pressure
LVEDV: Left ventricular end-diastolic volume
LVEF: Left ventricular ejection fraction
MAP: Mean arterial pressure
Na: Sodium
pCO_2_: Arterial partial pressure of carbon dioxide
pO_2_: Arterial partial pressure of oxygen
RU: Relative unit
S_a_O_2_: Arterial oxygen saturation
SAP: Systolic arterial pressure
S_cv_O_2_: Central venous oxygen saturation
SO_2_: Tissue oxygen saturation
SV: Stroke volume
TNF-α: Tumour necrosis factor-alpha
V–V: Veno-venous
